# Totality of Symptoms and Concomitance

**Published:** 1889-01

**Authors:** Stuart Close

**Affiliations:** Brooklyn, N. Y.


### TOTALITY OF SYMPTOMS AND CONCOMITANCE.

Stuart Close, M. D., Brooklyn, N. Y.

In all discussion, definition of terms is of the first importance. This is generally acknowledged, but often overlooked. Hence we too often see the pages of our journals filled with what might better have been left unsaid.

A correct understanding of certain words and phrases in common use would clear up all difficulty, and we should be spared the pain of reading unjust criticism, denunciation, and ridicule of men whose work has stood the test of continual and effective use for nearly a century.

These remarks are inspired by an article contained in the last issue of THE HOMŒOPATHIC PHYSICIAN (Dec., 1888), entitled "How to Find the Drug."

The line of argument adopted in this article leads to the conclusion that the qualities expressed by the words, "peculiar and uncommon," as applied by Hahnemann to symptoms, are *not* to be found in the symptoms themselves, but in their *concomitance*, and that *this* is essential to correct prescribing.

This conclusion is made to serve as a point from which to attack the Hahnemannian arrangement of the materia medica, which is denounced as "absolutely destroying this relation of concomitance," and ridiculed as being inextricably confused. What must be the inference as to its value, in the estimation of one who holds such views?

If such were the fact, it seems a little strange that so much could have been accomplished in the way of curing sick people since the days when Hahnemann gave his *Materia Medica Pura* to the world.

How shall we account for the position of our author, whose honesty and desire to arrive at the truth will not be questioned? Simply by pointing out his misapprehension of what a "symptom" really is, as manifested in his article.

On this point, which is of supreme importance, I quote from the inaugural address of President P. P. Wells before the International Hahnemannian Association in 1881, reprinted from the *Transactions*, in THE HOMŒOPATHIC PHYSICIAN, vol. I, p. 279.

These golden words should be studied, mastered, and kept continually in mind by every homœopathician, the more so as this misapprehension is very general:

"Let us see what is contained in the expression, '*totality of symptoms.*' As prescribers, it is with these we have our starting-point. Until we have these in our possession, we have no concern with the other factors of the problem we are about to try to solve. A right understanding of this fundamental expression is necessary before we can take the first step in a true homœopathic prescription. '*Totality of symptoms*'—what does it mean here? All the symptoms of the case, is it answered? It means this and more. The 'totality' here means not only the sum of the aggregate of the symptoms, but also this other and most important fact of all, in true homœopathic prescribing,

*the totality of each individual symptom of the aggregate group.* A SINGLE SYMPTOM IS MORE THAN A SIMPLE FACT; IT IS A COMPOUND, MADE UP OF A FACT, WITH ITS HISTORY, ITS ORIGIN, PROGRESS, AND CONDITIONS ATTACHED. If it be a cause of suffering to the patient, then in it are included all the circumstances of its aggravation or amelioration; as to the time of its greatest intensity, position, motion, rest; how affected by eating, drinking, or the performance of any bodily function; how affected, if at all, by different mental emotions; or by any other cause of increase or relief of suffering. All this is included in the '*totality*' of each single symptom, and without all this the prescriber is ignorant of the intimate nature of the symptom for which he is to find a simillimum."

A fact, then, *is not a symptom without all its concomitants.* The concomitants are an integral and necessary part of a symptom.

If, now, the symptoms of the *Materia Medica Pura* be examined in the light of this definition, it will at once be seen that they comply with the requirements. They stand out in symmetrical proportion, complete and rounded. There is no breaking up of the symptoms into fragments and scattering them here and there, as one would infer from the article under consideration, but they stand recorded, under their proper headings, exactly as they occurred in the prover. One would not naturally look for a symptom of the mind under the heading "Abdomen;" but if "*anxiety*" occurred in connection with "a sensation as if the abdomen were hollow," *you will find it so stated.* (*Vide Materia Medica Pura*, article Chamomilla, symptom 183.) This symptom may serve as an illustration of the truly Hahnemannian method. Of its ignorant and incompetent imitators nothing need be said.

The symptom referred to reads as follows (Dudgeon's translation): "Sensation as if the whole abdomen were hollow, and at the same time a perpetual movement in the bowels (with blue rings around the eyes), and when the attack comes on in the evening, it is for a short time combined with anxiety." We have here the statement of a fact, with its elements and concomitants of time, place, and circumstances, and all these together make up one, and only one, symptom, which is classified, for convenience of reference, under the general heading of "Abdomen."

The article under discussion would lead us to infer that Hahnemann's plan would not only separate the several elements of which this symptom is composed, but would scatter them about under wholly arbitrary and inappropriate heads, to the total confusion of the true drug-picture. In the elegant phraseology of the article, "Instead of getting a clear picture of the drug-sickness as actually caused by that drug, the beginning is placed at the end, the middle is at the beginning or anywhere else, and the end comes first or middle or wherever chance falls."

Simple reference to the *Materia Medica Pura* of Hahnemann is all that is needed to confute such a statement.

